# The role of alcohol use and adiposity in serum levels of IL-1RA in depressed patients

**DOI:** 10.1186/s12888-022-03784-8

**Published:** 2022-03-02

**Authors:** Mari Archer, Onni Niemelä, Mari Hämäläinen, Eeva Moilanen, Esa Leinonen, Olli Kampman

**Affiliations:** 1 Tampere University, Faculty of Medicine and Health Technology, P.O. Box 100, 33014 Tampere, Finland; 2 Department of Laboratory Medicine and Medical Research Unit, Seinäjoki Central Hospital and Tampere University, 60220 Seinäjoki, Finland; 3 The Immunopharmacology Research Group, Tampere University, Faculty of Medicine and Health Technology, and Tampere University Hospital, 33014 Tampere, Finland; 4 Tampere University, Faculty of Medicine and Health Technology, P.O. Box 100, 33014 University of Tampere, and Tampere University Hospital, 33014 Tampere, Finland; 5 Tampere University, Faculty of Medicine and Health Technology, P.O. Box 100, 33014 University of Tampere and Pirkanmaa Hospital District, Department of Psychiatry, 33521 Tampere, Finland

**Keywords:** Mood disorder, Cytokine, Ethanol, Obesity

## Abstract

**Background:**

The role of Interleukin-1 Receptor antagonist (IL-1Ra), an innate antagonist to pro-inflammatory cytokine IL-1, has attracted increasing attention due to its potential pathogenic and therapeutic implications in depression. However, the role of alcohol and adiposity in modulating IL-1Ra cytokine pathway in depressed patients has remainned unknown. The aim of this study was to follow the changes in IL-1Ra serum levels in depressed patients with or without simultaneous alcohol use disorder (AUD) and different degrees of adiposity during 6 months of follow-up.

**Materials and methods:**

A total of 242 patients with depression were followed for 6 months. At baseline 99 patients had simultaneous AUD. Levels of serum IL-1Ra and common mediators of inflammation (IL-6, hs-CRP) were measured. Clinical assessments included Body Mass Index (BMI), Montgomery-Asberg Depression Rating Scale (MADRS) and Alcohol Use Disorders Identification Test (AUDIT) scores.

**Results:**

Significant reductions in clinical symptoms and IL-1Ra were observed during 6-month follow-up. In hierarchical linear regression analysis, the effect of MADRS score, age, gender, and smoking had a combined effect of 2.4% in the model. The effect of AUDIT score increased the effect to 4.2% of variance (*p = 0.08*), whereas adding BMI increased the effect to 18.5% (*p <  0.001*).

**Conclusion:**

Adiposity may influence the IL-1Ra anti-inflammatory response in depressed patients, whereas the effect of alcohol consumption in these patients seems insignificant. These findings should be considered in studies on the role of IL-1Ra in depression.

**Trial registration:**

Ostrobothnia Depression Study in ClinicalTrials.gov , Identifier NCT02520271.

## Background

Major depressive disorder (MDD) is one of the leading causes of disability worldwide (WHO), and accumulating evidence suggests participation of activated immune-inflammatory pathways in the onset of depression [1–3]. Several studies have reported elevated levels of inflammatory mediators in patients with depression, as summarized in a recent review by Köhler et al. [4], and in translational models the administration of pro-inflammatory cytokines has been shown to induce depression-like symptoms [5]. Also, a persistent inflammatory state has been suggested to have a role in treatment-resistant depression [6, 7].

Interleukin I (IL-1) is a crucial mediator of the inflammatory response, playing an important role in the development of pathological conditions leading to inflammation. Currently a total of 11 members of the IL-1 family have been indentified, most importantly pro-inflammatory cytokines IL-1α and IL-1 β [8, 9]. IL-1 receptor antagonist (IL-1Ra) is synthesized and released in response to the same stimuli as IL-1, as a compensatory mechanism to attenuate inflammatory reaction [10]. Binding of IL-1Ra to IL-1 receptor inhibits signaling of IL-1, making IL-1Ra one of the most potent anti-inflammatory mediators [11].

Several studies have indicated significant associations between elevated serum IL-1 [12, 13] as well as IL-1Ra and depression [14–17]. Additionally, levels of IL-1 have been suggested to increase or to show failure to normalize in patients with persistent depressive symptoms despite medical treatment [15, 18–20]. Elevated levels of IL-1Ra have also been found in patients with treatment-resistant depression [15], but this association has not been confirmed.

Although alcohol use disorder (AUD) is an extremely common co-morbidity with depression [21], the question whether excessive alcohol drinking affects levels of IL-1Ra and patient recovery in depressed patients has remained unknown. Previous studies have reported compensatory increase in levels of IL-1Ra in response to pro-inflammatory state produced by AUD in pulmonary disease [22], and a potential use of IL-1Ra recombinant in the treatment of alcohol induced liver disease has been suggested [23]. However, a recent study by Bjorkhaug et al. [24] found no significant changes in levels of IL-1Ra in men with AUD, and these authors concluded that patients with AUD showed a cytokine profile suggestive of increased overall pro-inflammatory activity.

Adipose tissue is a substantial source for IL-1Ra secretion [25], and obesity has been shown to correlate with increased peripheral levels of IL-1Ra [24, 26]. In addition, cytokines in depressed patients are commonly expressed in a gender-dependent manner [27]. Although a large, population-based study recently reported elevated levels of IL-1Ra in males with depressive symptoms [28], the role of gender in the expression of IL-1Ra in psychiatric disorders has remained elusive.

While a putative role of IL-1Ra in depression has been suggested, there is inconsistency in findings of serum IL-1 or IL-1Ra levels in depressed patients with or without comorbidities such as alcohol use disorder (AUD), or with differing degrees of adiposity. Therefore, we explored changes in serum levels of IL-1Ra during a six-month follow-up of depressed patients with or without simultaneous AUD and with or without excess body weight.

## Methods

### Study design and participants

Patients were recruited between 2009 and 2013 from five psychiatric outpatient clinics and from one Finnish psychiatric hospital in the South Ostrobothnia Hospital District (population 200,000), in total 242 participants. Patients were suffering from depressive symptoms and possible co-morbid anxiety, self-destructiveness, insomnia or alcohol or other substance related problems.

Inclusion criterion for the study was clinical depression. The depressive state of all patients was assessed with the Beck Depression Inventory (BDI, version 1A). Patients with a BDI score ≥ 17 at the screening phase were consequently considered to be at least moderately depressed and were included in the study [29]. Exclusion criteria were primary diagnosis of psychotic disorders (ICD-10 codes F20–29), or organic brain disease or damage. The study was approved by the local ethics committee and all participants provided written informed consent. The study protocol is outlined and published at Ostrobothnia Depression Study in ClinicalTrials.gov, Identifier NCT02520271; first posted date 11/08/2015, and by Archer et al. [30]. The study protocol is described in detail: https://www.clinicaltrials.gov/ct2/show/NCT02520271?term=ODS&draw=2&rank=1.

At baseline, The Mini International Neuropsychiatric Interview 5.0 (MINI) diagnosis was available for 219 patients. However, all patients in this study were classified as depressed when evaluated with the BDI questionnaire. The baseline assessment for all patients included BDI-21, Montgomery-Åsberg Depression Rating Scale (MADRS), AUDIT-10 (Alcohol Use Disorders Identification Test) and additional questions about alcohol use following a timeline follow-back method (amounts per week, duration of harmful drinking). Patients also completed up a questionnaire on substance use other than alcohol from the past 12 months prior to the study. Since the number of patients with co-morbid substance use was relatively small (twenty patients in the AUD group and two patients in the non-AUD group), this was not chosen as an exclusion criterion for the study. Smoking status was also recorded based on patient self-reporting.

The clinical assessment included measurement of body mass index (BMI), and waist circumference to the nearest 0.5 cm obtained from the measurements between the lowest rib and iliac crest while the study subject was at minimal respiration. The clinical assessments were performed by a psychiatrist or a research nurse with long experience in the field of psychiatry and appropriate methodological training.

Participants were divided into two groups according to the baseline AUDIT-score, where patients with ≥11 AUDIT points were categorized as MDD + AUD. The WHO guidelines for AUDIT use in primary care [31] state that selection of cut-off point should be dependent on national and cultural standards, and in Finnish clinical practice with AUD patients, AUDIT score ≥ 11 points is considered to indicate significantly increased risk of harmful alcohol consumption. A concordance of 88% in AUDIT score and substance use disorder assessed with M.I.N.I. has been previously reported in this patient sample [32], confirming reliability of AUDIT score as an indicator of alcohol use disorder. All data were analyzed between study groups and between genders, where gender groups were formed based on the sex assigned at birth.

The selection of the therapeutic treatment intervention was based on the baseline levels of alcohol use measured with AUDIT score and included behavioral activation therapy for all patients of this study and, additionally, motivational interview (MI) for patients with co-morbid alcohol use disorder (AUD). Use of medication was based on psychiatrist’s estimation, and the study protocol did not guide the choice of the pharmaceutical agent. Nonetheless, evaluation of the effectiveness and possible adjustments of medication were recommended when MADRS score exceeded 19 points at baseline. Data on medications taken before entering this study was not available. However, all patients were referred to psychiatric specialty unit following initial treatment period in general health services following the recommendations given in the Current Care Guidelines of Finland, with a requirement of medication attempt prior to referral. For statistical purposes all anti-depressant medications were converted-to equivalent doses of fluoxetine, and anti-psychotic medications to equivalent doses of chlorpromazine. Data on medication adherence was acquired from the medical records and by keeping paper-and-pencil diaries during the first 6 weeks of the study.

The number of visits during the study period varied based on the requirements of care for each patient. Six-month follow-up appointment included a visit to a research nurse, which included the assessment of the severity of depression with MADRS score and gathering information on alcohol use using the AUDIT questionnaire and analyses of blood biomarkers. Also, weight and waist circumference were measured, and BMI calculated.

### Laboratory assays

Venous blood samples were drawn from each subject at baseline and at 6 months. The patients did not have specific appointment to the laboratory, but due to the operating hours of the regional laboratory services, the samples were obtained in the morning. Serum was separated by centrifugation and stored at − 80 °C prior to analysis. Serum IL-1Ra concentration was measured by Quantikine high sensitivity enzyme-linked immunosorbent assay (ELISA) (R&D Systems Europe, Abingdon, UK). The detection limit and the interassay coefficient of variation were 15.6 pg/ml and 7.4%, respectively. High Sensitivity CRP (hs-CRP) was measured using particle-enhanced immunoturbidimetric method on an automated clinical chemistry analyzer (Roche Diagnostics, Basel, Switzerland). Concentration of serum IL-6 was determined using Quantikine high sensitivity ELISA kit according to the instructions of the manufacturer (R&D Systems, Abington Science Park, UK).

### Statistics

The data are reported as mean ± SD. The laboratory parameters showed a tendency towards lognormal distributions, although meeting the limits of normality, with the exception of 2–8 outliers per studied variable. To obtain non-skewed distributions with homogeneity of variance the data were transformed into + 2.5 SD levels as previously described by Miller [33]. Q-Q plots were drawn for all experimental laboratory tests, showing normality of distributions.

Comparison of means between genders and between patients with depression or depression + AUD were analyzed with independent samples *t*-test at baseline and after 6 months of treatment.

To analyze the association of baseline IL-1Ra levels and confounding factors hierarchical linear regression analyses were performed. The model was adjusted for MADRS score, age, gender, and regular smoking, whereas the effect of baseline AUDIT score was added at step two and BMI at step three of the analysis.

A linear mixed model for repeated measures was used to predict changes in IL-1Ra during the follow-up from baseline to 6 months with various possible confounding factors (gender, AUD status, smoking, severity of MDD estimated by MADRS score, age, BMI, and interaction terms time*AUD, and time *MADRS).

Pearson’s correlation coefficient was used to calculate correlations between known inflammatory biomarkers IL-6 and hs-CRP and anti-inflammatory IL-1Ra at baseline.

## Results

### The main characteristics of the study population are summarized in Table 1

A significant reduction of depressive symptoms was observed in all study groups during six-month follow-up (*p* <  0.001 for men and women, AUD and non-AUD). BMI increased in all study groups during the follow-up period, + 1.8% in women and + 3.3% in men (*p* <  0.05); + 1.2% in non-AUD and + 4.3% in AUD groups (*p* <  0.05).

**Table 1:** Main demographic characteristics of the patients

	All	Women	Men	Non-AUD	AUD
n1	242	148	94	143	99
n2	148	93	55	101	47
Drop-out rate	−39%	−37%	−41%	−29%	−53%
Age, years (range)	38.8 (17–64)	38.1 (17–64)	39.9 (18–64)	38.7 (17–64)	38.9 (18–62)
MADRS, baseline (SD)	23.16 (6.70)	22.26^a^ (6.79)	24.52 (6.37)	22.83^b^ (6.73)	23.65 (6.67)
MADRS, 6 months (SD)	13.26 (8.72)	11.72^c^ (8.23)	15.85 (9.00)	12.87^d^ (8.69)	14.09 (8.83)
Effect of treatment in MADRS score	*p <* 0.001	*p <* 0.001	*p <* 0.001	*p <* 0.001	*p <* 0.001
Alcohol doses /week, mean (SD)	10.1 (22.8)	5.5 (9.2)	16.9 (32.9)	1.9 (3.9)	20.4 (31.2)
Median (IQR)	1.0 (12.0)	1.0 (8.0)	25 (19.0)	0 (2.0)	12.0 (25.0)
BMI, baseline (SD)	28.22 (6.67)	28.49 (7.39)	27.80 (14.36)	28.46 (7.08)	27.86 (6.03)
BMI, 6 months (SD)	28.89 (6.74)	29.01 (7.60)	28.71 (5.19)	28.81 (7.27)	29.07 (5.53)
Cigarettes per day (SD)	8.30 (9.61)	6.49 (6.49)	11.26 (11.26)	5.38 (8.25)	12.78 (9.88)
n3	206	120	86	130	76
Flxeqv/mg per day (SD)	28.0 (20.6)	25.0 (18.6)	32.7 (21.3)	26.0 (18.9)	30.9 (22.5)
n4	66	33	33	27	39
Cpzeqv/mg per day (SD)	111.1 (129.3)	108.8 (140.0)	119.8 (113.4)	91.2 (90.0)	124.8 (150.3)

n1, number of patients at baseline; n2, number of patients at 6 months; n3, number of patients with anti-depressant medication; n4, number of patients with anti-psychotic medication.

Flxeqv, fluoxetine equivalent dose; Cpzeqv, chlorpromazine equivalent dose

Drop-out rate including patients with full symptom recovery and treatment conclusion before 6-month follow-up. Significance of difference in drop-out rate between study groups assessed with Pearsons Chi-Square test (*χ*^2^ = 14.04, df = 2, *p* = 0.001).

^a^ Mean difference in MADRS score between men and women at baseline *p* < 0.05; ^b^ Mean difference in MADRS score between AUD and non-AUD groups at baseline *p* = *ns*; ^c^ Mean difference in MADRS score between men and women at 6 months *p* < 0.01; ^d^ Mean difference in MADRS score between AUD and non-AUD groups at 6 months *p* = *ns*

### Effect of gender and AUD on levels of serum IL-1Ra

The levels of IL-1Ra changed during the six-month follow-up in all study groups, the mean concentration of all groups combined at baseline was 764 pg/ml, and at six-months 670 pg/ml (*p = 0.05*).

At baseline, the effect of gender on the levels of IL-1Ra was insignificant (*t = 1.745; 95%CI -20.67 – 339.46; p = 0.08*), as was in cases with alcohol use (*t = 0.008; 95%CI -180.24 – 181.74; p = 0.99*). After 6 months of treatment, the effect of gender and alcohol use remained insignificant (*t = 0.881; 95%CI -97.09 - 252.74; p = 0.38* for gender, and *t = 1.03; 95%CI -85.80 – 273.57; p = 0.30* for alcohol use).

### Association of severity of depression, smoking, adiposity, and excessive alcohol use on levels of serum IL-1Ra at baseline

Hierarchical linear regression analysis was used to assess factors affecting levels of IL-1Ra at baseline. The effect of MADRS score, age, gender, and regular smoking was adjusted, having a combined effect of only 2.4% in the model (*F = 0.96; p = 0.43*). The effect of AUDIT score increased the effect to 4.2% of variance (*F = 3.08; p = 0.08*). Adding BMI into the model increased the effect to 15.3% of variance (*F = 27.38; p <  0.001*). These results are presented in detail in Table 2.

**Table 2:** Hierarchical regression analysis for the effect of confounding factors on levels of IL-1Ra at baseline. Effect of all confounding factors: *R*^*2*^ adjusted = 0.15, *p* < 0.001

Dependent variable: IL-1Ra	Estimate (*B*)	95% CI lower	95% CI Upper	*β*	*t*	*p*
Explaining variables:						
Age	−3.63	− 12.46	5.19	− 0.06	−0.81	0.42
Gender	− 215.80	− 444.24	12.66	−0.17	−1.97	0.05
MADRS score	−80.10	− 311.86	151.66	−0.06	−0.68	0.50
Regular smoking	−71.93	− 304.26	160.39	−0.05	−0.61	0.54
AUDIT score	11.87	−0.28	24.02	0.16	1.93	0.08
BMI	44.25	27.55	60.96	0.39	5.23	< 0.001

### Association of confounding factors with changes in the serum IL-1Ra levels during 6-months follow-up

In the linear mixed model for repeated measures analysis, we found levels of IL-1Ra to decrease during the study period (*F = 10.38; p = 0.002*). The effect of gender and smoking was insignificant, but adiposity at baseline (*F = 46.99; p <  0.001*) and age (*F = 145.45; p = 0.036*) had a significant effect on levels of IL-1Ra at 6 months follow-up. The effect of the interaction term time*MADRS was also significant (*F = 8.11; p = 0.005*). These results are presented in detail in Table 3.

**Table 3:** Results of linear mixed model for repeated measures in predicting the changes in IL-1Ra levels from baseline to 6-month follow-up. The explaining variables in all models were gender and age as confounding factors, time from baseline to 6 months, regular smoking, alcohol use (AUD/non-AUD), depression severity at baseline in MADRS scores, and combined effects of time*AUD and time*MADRS. Estimates of factors gender, AUD, time, and time*AUD are for the following subgroups: female gender, non-AUD, baseline and the interaction of baseline*non-AUD. Interpretation of factor estimates accordingly: a negative estimate indicates lower biomarker levels in women or in non-AUD group at baseline. The combined effect of time*AUD and time*MADRS represents the effect of given variable (AUD / MADRS) on the change in the levels of IL-1Ra over time

Dependent variable: IL-1Ra change in 0–6 months	Estimate	95% CI	SE	*t*	*p*
Explaining variables:					
Gender	128.55	−45.51 – 302.61	88.07	1.46	0.15
Age	−6.98	−13.52 – –0.45	3.31	−2.11	0.04
Time	397.71	200.18–595.23	99.94	3.98	< 0.001
Regular smoking	35.84	−129.76 – 201.44	83.74	0.43	0.67
AUD	121.83	−71.90 – 315.57	97.93	1.24	0.22
MADRS	186.03	21.35–350.71	83.16	2.24	0.03
BMI	42.40	30.16–54.63	6.19	6.86	< 0.001
Time*AUD	− 183.42	−387.80 – 0.95	103.38	−1.77	0.08
Time*MADRS	− 279.49	− 473.57 – –85.41	98.15	−2.85	0.005

### Correlation of serum IL-1Ra with inflammatory biomarkers IL-6 and hs-CRP

A significant correlation of IL-1Ra and IL-6 at baseline was observed in women (r = 0.45; *p* < 0.001), in the non-AUD group (*r = 0.42; p < 0.001*) and in the AUD group (*r = 0.31; p = 0.004*), but not in men (*r = 0.20; p = 0.07*).

The correlation of IL-1Ra and hs-CRP at baseline was significant in women (*r = 0.23; p = 0.01*), men (*r = 0.22; p = 0.05*) and non-AUD (*r = 0.29; p = 0.01*) but not in the AUD group (*r = 0.17; p = 0.13*).

## Discussion

Our study including 242 depressed patients indicates significant reductions in both depressive symptoms and levels of IL-1Ra during a six-month follow-up. The effect of AUD remained insignificant in relation to serum levels of IL-1Ra, whereas levels of IL-1Ra were found to be affected by adiposity.

Previous studies have emphasized the need for novel biomarkers to help detect processes in recovery of depression. All patients in this study were suffering from clinically significant depression, and we have previously reported a decrease in the depressive symptoms in all study groups during the 6 months follow-up [30]. We have also previously observed elevated serum levels in biomarkers of inflammation in depressed patients, and that the levels of these biomarkers appear to decrease during treatment [30].

Excessive ethanol consumption has been shown to play a key role in altering levels of cytokines and other mediators of inflammation in a variety of tissues [34, 35]. A recent review and meta-analysis by Adams et al. [36], reported increased concentrations of cytokines in AUD patients when comparing to healthy control. Furthermore, the levels of circulating cytokines have been found to be affected by the stage of illness [36]. Also, IL-1 receptor signaling has recently been suggested as an important player in the development of alcohol dependence, and as a key regulator of various neuroimmune responses [37]. Research has indicated elevated IL-1 and IL-1Ra levels in response to alcohol consumption [38], and Neupane et al. [39] have presented an experimental study where binge drinking induced elevated IL-1Ra levels in healthy volunteers. In light of previous studies, the assumption regarding the effect of AUD on IL-1Ra levels was justifiable, yet, based on our findings, one might suggest that anti-inflammatory response to alcohol consumption could be altered in this patient population. To the best of our knowledge, the present study is the first to examine changes in serum IL-1Ra levels in light of alcohol consumption in patients with depression, a clinically important subgroup of depressed population. When the correlation of IL-1Ra with other inflammatory biomarkers was compared between the AUD and non-AUD groups of this study, there was a strong correlation between serum levels of IL-1Ra and IL-6 in the non-AUD but not in the AUD group, thereby supporting the assumption that alcohol may modulate the mechanisms regulating the balance between pro- and anti-inflammatory response in depressed patients.

A wide array of previous studies have presented the inflammatory effect of adipose tissue in patients with depression [40, 41], and adipose tissue has also been reported to function as a major source of commonly known pro-inflammatory biomarkers such as IL-6 [42]. Therefore, the levels of IL-1Ra may rather express the ongoing inflammatory condition in depressed patients, than demonstrate the direct relation of increased adiposity.

Since smoking or alcohol use had no direct effect on serum IL-1Ra levels, we utilized linear mixed model to examine factors contributing to the change, using the combined effect of follow-up time and detected AUD as well as the effect of follow-up time and MADRS to represent the effects of a given variable on changes in levels of serum IL-1Ra over time. Gender and smoking did not affect the levels of IL-1Ra, but body adiposity had a significant effect, which corroborates existing knowledge of adipose tissue as a significant source of IL-1Ra. Even in this analysis, AUD or the combined effect of time and AUD had no significant effect on the change in IL-1Ra serum levels, whereas the severity of depressive symptoms measured as MADRS scores, and its interaction with the time variable had a significant effect.

The role of IL-1Ra in depression has recently attracted growing interest together with the development of anti-cytokine medications. It is widely acknowledged that only a part of depressed patients benefit from the present anti-depressive medications, and these findings have led to novel theories of treating depression with anti-inflammatory agents. A recent meta-analysis of the effects of anti-cytokine treatments for depressive symptoms by Kappelmann et al. [43] suggested improvement of depressive symptoms in patients treated with anti-cytokine agents adalimumab, etanercept, infliximab and tocilizumab, independent of changes in primary physical condition. In a study by Raison et al. [44], anti-TNF-α antibody infliximab was shown to have a significant effect on decreasing depressive symptoms in patients previously not responding to medication. IL-1Ra recombinant anakinra has been recently suggested as a novel biological response modifier drug for autoimmune inflammation [45] and the importance of controlling the effects of pro-inflammatory IL-1 with this molecule has also been suggested in psychiatric morbidities [46]. Determining and identifying potential subgroups of patients benefiting from this targeted treatment requires further investigation.

### Strengths and limitations

The strength of this study lies in the naturalistic setting, with an interface to the clinical reality faced by psychiatrists. We assessed a clinically relevant dilemma in depression, where it is still widely unknown which patients are resistant to treatment, and what biological mechanisms could explain this. Substance use has hitherto been an exclusion criterion in many studies on depression, yet in this study we have specifically aimed to assess the role of alcohol consumption in biochemical changes observed in depression. Also, presenting longitudinal data, as we have done in this report, adds valuable information to the literature on depression.

The limitation of this study is the absence of a healthy, non-depressed study group. Nevertheless, this should not compromise the conclusions: the study parameters have been separately studied in several earlier reports. The extent of alcohol consumption was assessed by methods relying on self-evaluation. Yet we have previously reported a significant correlation between self-reports and blood biomarkers of alcohol use in this study sample and can therefore confirm the accuracy in the assessment of alcohol use [47].

## Conclusions

Our data suggests that effects of adiposity markedly influences IL-1Ra anti-inflammatory response in patients with depression, whereas the direct immunomodulatory effect of alcohol consumption on IL-1Ra appears limited.

## Data Availability

The datasets generated and analyzed during the current study are not publicly available due to data containing sensitive information but are available from the corresponding author on reasonable request.

## Abbreviations

AUD: Alcohol Use Disorder
AUDIT: Alcohol Use Disorders Identification Test
BMI: Body Mass Index
hs-CRP: High Sensitivity C-reactive Protein
ICD-10: International Statistical Classification of Diseases and Related Health Problems 10
IL-1: Interleukin 1
IL-1Ra: Interleukin 1 Receptor Antagonist
IL-6: Interleukin 6
MADRS: Montgomery Åsberg Depression Rating Scale
MDD: Major Depressive Disorder
MI: Motivational Interview
MINI: The Mini International Neuropsychiatric Interview
TNF- α: Tumor Necrosis Factor Alpha
WHO: World Health Organization
